# The analysis of corneal asphericity (Q value) and its related factors of 1,683 Chinese eyes older than 30 years

**DOI:** 10.1371/journal.pone.0176913

**Published:** 2017-05-18

**Authors:** Ying Xiong, Jing Li, Ningli Wang, Xue Liu, Zhao Wang, Frank F. Tsai, Xiuhua Wan

**Affiliations:** 1 Beijing Tongren Eye Center, Beijing Tongren Hospital, Capital Medical University, Beijing, China; 2 Beijing Institute of Ophthalmology, Beijing Tongren Hospital, Capital Medical University, Beijing, China; 3 Beijing Key Laboratory of Ophthalmology and Visual Science, Capital Medical University, Beijing, China; 4 Sharp Rees-Stealy Medical Group, San Diego, CA, United States of America; Universita degli Studi di Firenze, ITALY

## Abstract

**Purpose:**

To determine corneal Q value and its related factors in Chinese subjects older than 30 years.

**Design:**

Cross sectional study.

**Methods:**

1,683 participants (1,683 eyes) from the Handan Eye Study were involved, including 955 female and 728 male with average age of 53.64 years old (range from 30 to 107 years). The corneal Q values of anterior and posterior surfaces were measured at 3.0, 5.0 and 7.0mm aperture diameters using Bausch & Lomb Orbscan IIz (software version 3.12). Age, gender and refractive power were recorded.

**Results:**

The average Q values of the anterior surface at 3.0, 5.0 and 7.0mm aperture diameters were -0.28±0.18, -0.28±0.18, and -0.29±0.18, respectively. The average Q value of the anterior surface at the 5.0mm aperture diameter was negatively correlated with age (B = -0.003, p<0.01) and the refractive power (B = -0.013, p = 0.016). The average Q values of the posterior surface at 3.0, 5.0, and 7.0mm were -0.26±0.216, -0.26±0.214, and -0.26±0.215, respectively. The average Q value of the posterior surface at the 5.0mm aperture diameter was positively correlated with age (B = 0.002, p = 0.036) and the refractive power (B = 0.016, p = 0.043).

**Conclusion:**

The corneal Q value of the elderly Chinese subjects is different from that of previously reported European and American subjects, and the Q value appears to be correlated with age and refractive power.

## Introduction

The cornea is the major refractive component in a human eye, contributing roughly 70% of the total refractive power. Previous studies found that the cornea could be described as a quadric surface with surface asphericity [1–3]. The Q value, a quantified indicator of the aspherical degree, is defined as radial change from center to peripheral of the quadric surface. As a key parameter of the mathematical model of the cornea, the Q value reflects the corneal shape and optical properties [4,5] including refractive power, spherical aberration, aberration distribution, etc. More recently, research has focused on the study of the corneal Q value and its distributions, as well as its influence on optical properties of the human eye [6–9]. Although the corneal Q value in the elderly population is an important factor in the design of intraocular lens (IOL) and for the treatment of refractive errors [10,11], there are few formal studies evaluating Chinese elderly subjects. This study aims to determine the corneal Q value of the elderly Chinese population and its distribution, which may aid in a more accurate model for the Chinese eye and IOL.

## Method

### Study population

The Handan Eye Research [12] looked at common eye diseases by cross-sectional mapping. A total of 8,653 Chinese subjects over the age of 30 years old were enrolled. Eyes with any corneal disease or pathology were excluded. No subjects underwent the LASIK surgery, nor used contact lenses. Corneal topography of the right eye of 2,958 subjects was measured. Final analysis was performed on 1,683 subjects, including 995 females and 728 males, after excluding eyes with poor measurement repeatability, incomplete topographic map, or high deviation from the reference surface. The average age was 53.64 ± 11.02 years old (range 30–107) and average spherical equivalent power was 0.81 ± 1.50D (range -11.87–+5.87D). The Study protocol was approved by the ethical committee of Beijing Tongren Hospital. The study adhered to the tenets of the Declaration of Helsinki. Written informed consent was obtained from each subject after the purpose of the study and protocols had been explained to all participants.

### Method

Corneal topography measurement was repeated twice for each of 2,958 subjects’ right eye using Bausch & Lomb Orbscan IIz (software version 3.12). Data were analyzed for the full topographic map measurements at aperture diameter greater than 7.0mm. During the analyses, the measurements with poor repeatability (difference in Q value greater than 0.7 between two measurements) or having large dark purple or red areas (high deviation from the reference surface) were excluded. The Q value at 3.0, 5.0 and 7.0mm aperture diameters was calculated by the Pachymetry Stats program, a built-in software of the Orbscan IIz which applied the Mean Fit Method (fitting the corneal map surface to the reference surface) in calculation. The average Q values of the two repeated measurements were used as the final Q value for statistical analysis. The results included the mean corneal Q values (mean ± SD) of the anterior and posterior surfaces at different aperture diameters, the Q value distributions of the anterior and posterior surfaces at large aperture (7.0mm diameter), and analysis of the relationship between the Q values and age, sex and refractive powers.

### Statistical analysis

Data were analyzed using SPSS software (version 18.0.0). Descriptive statistics was used to calculate average Q value (mean ± SD) for different aperture diameters, age, sex and refractive powers. T-test was performed to compare Q values between the male and female groups. Variance analysis was performed to compare the groups with different aperture diameters, age and refractive powers. Multiple regression analysis was used to explore the relationship between corneal Q value and age, sex and refractive power. We considered a p-value of ≤0.05 to be statistically significant.

## Results

### Subject's age distribution

The final 1,683 subjects’ mean age was 53.64 ± 11.02 years old (range 30–107). The subjects from age 30 to 39 accounted for 12.3%, 40 to 49 accounted for 17.9%, 50 to 59 accounted for 41.5%, 60 to 69 accounted for 20.4%, 70 years old and above accounted for 7.9% of the study population. Senile cataract generally develops after the age of 50, and in this study 50 years old and above accounted for 69.8%.

### Corneal Q values

The mean corneal Q values of the anterior surface were: -0.28(±0.18), -0.28(±0.18) and -0.29(±0.18) at 3.0, 5.0 and 7.0mm aperture diameters, respectively. The mean corneal Q values of the posterior surface were: -0.26(±0.22), -0.26(±0.21) and -0.26(±0.22) at 3.0, 5.0 and 7.0mm aperture diameters, respectively (Table 1).

**Table 1:** Corneal Q values.

Aperture Diameter (mm)	Corneal Q Value Anterior Surface	Corneal Q Value Posterior Surface
**3.0**	-0.28±0.18	-0.26±0.22
**5.0**	-0.28±0.18	-0.26±0.21
**7.0**	-0.29±0.18	-0.26±0.22
**F**	0.180	0.056
**P**	0.835	0.945

### Corneal Q value distribution at large aperture diameter of 7.0mm

**Fig 1:**
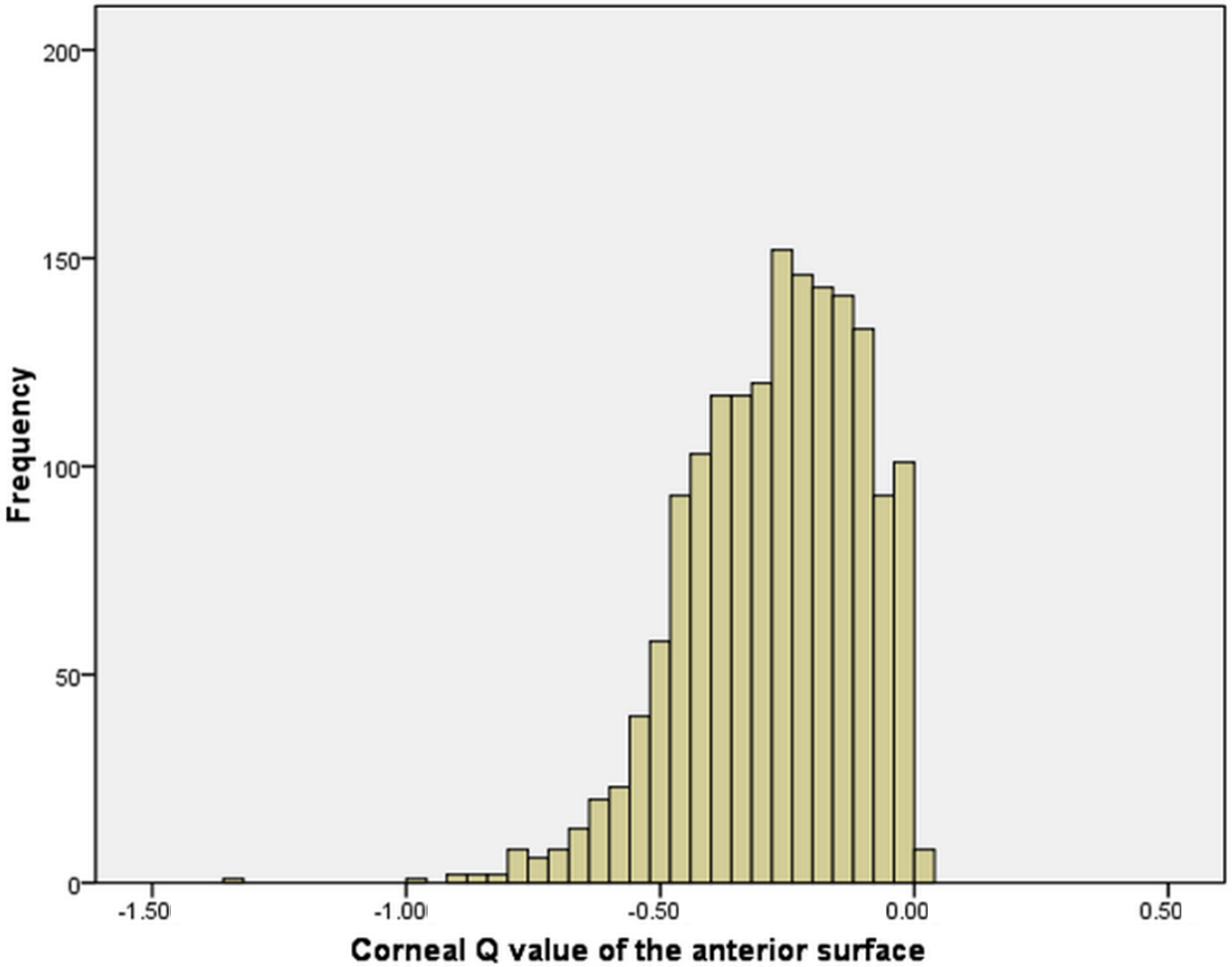
Corneal Q value distribution of the anterior surface.

**Fig 2:**
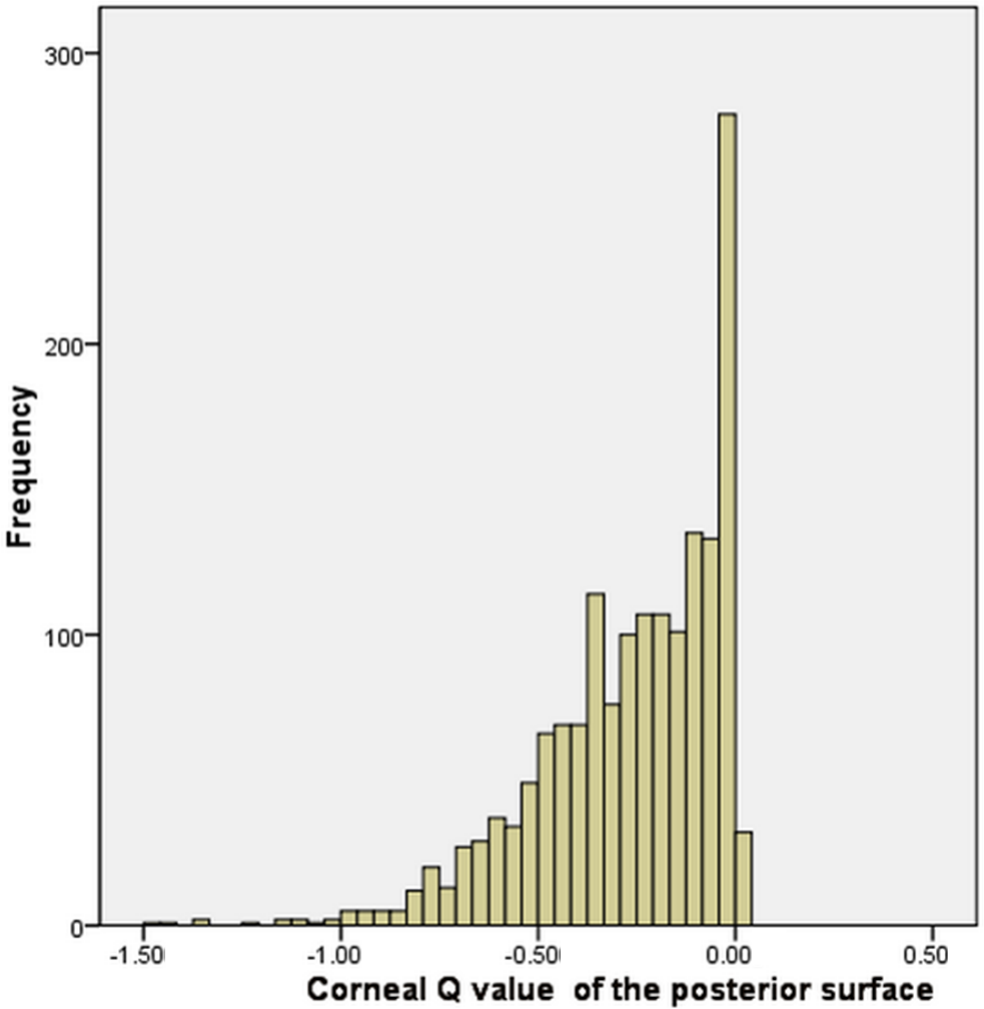
Corneal Q value distribution of the posterior surface.

The mean Q value of the anterior surface was -0.29 ± 0.18 (range -0.30 to -0.28, CI 95%, Fig 1), and for the posterior surface was -0.26 ± 0.21 (range -0.28 to -0.26, CI 95%, Fig 2).

### Corneal Q values for different age groups

**Fig 3:**
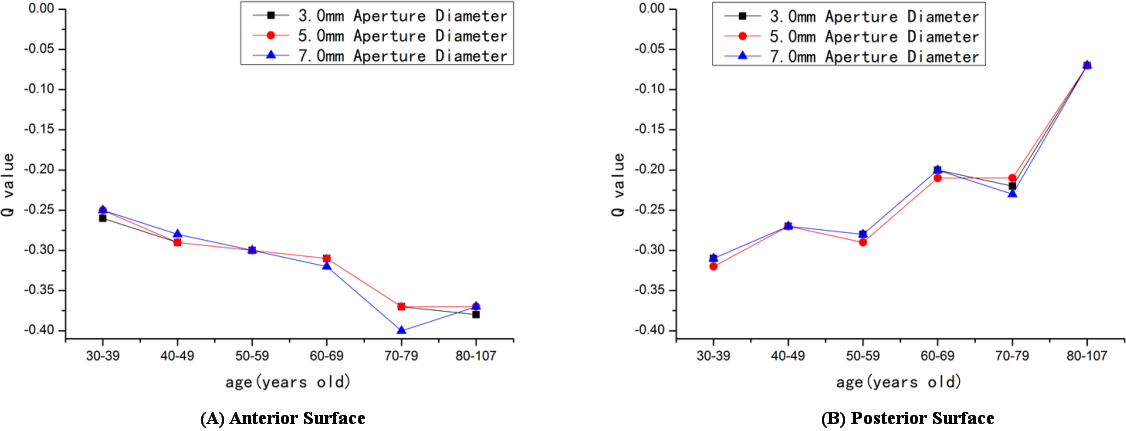
The trend lines of the Q value according to age and the opening diameters. (A) Anterior surface. (B) Posterior surface.

The distribution of Q value at different age stages is summarized in Table 2. The Q values of the anterior surface at 7.0mm aperture diameter and the posterior surface at 3.0, 5.0 and 7.0mm were statistically significant (*p*<0.05) across the different age groups. The corneal Q value of the anterior surface decreased with increasing age. However, the Q value of the posterior surface increased with increasing age (Fig 3).

**Table 2:** Corneal Q values for different age groups.

Aperture Diameter (mm)	Age Group (years old)						F	p
	30–39	40–49	50–59	60–69	70–79	80–107		
**3.0 (Anterior)**	-0.26±0.16	-0.29±0.16	-0.30±0.19	-0.31±0.19	-0.37±0.20	-0.38±0.17	1.60	0.157
**3.0 (Posterior)**	-0.31±0.20	-0.27±0.21	-0.28±0.23	-0.20±0.21	-0.22±0.21	-0.07±0.04	4.49	0.000
**5.0 (Anterior)**	-0.25±0.17	-0.29±0.16	-0.30±0.19	-0.31±0.20	-0.37±0.20	-0.37±0.18	1.81	0.109
**5.0 (Posterior)**	-0.32±0.21	-0.27±0.20	-0.29±0.23	-0.21±0.21	-0.21±0.20	-0.07±0.04	4.78	0.000
**7.0 (Anterior)**	-0.25±0.16	-0.28±0.16	-0.30±0.19	-0.32±0.19	-0.40±0.19	-0.37±0.16	2.78	0.017
**7.0 (Posterior)**	-0.31±0.21	-0.27±0.20	-0.28±0.22	-0.20±0.21	-0.23±0.20	-0.07±0.05	4.25	0.001

### Corneal Q values for male and female groups

Table 3 shows the mean Q values (mean ± SD) calculated for male and female groups at different aperture diameters. The Q values of the anterior surfaces at 3.0, 5.0 and 7.0mm aperture diameters were statistically significant (*p*<0.05) between the male and the female groups, while the Q values of the posterior surface were not significant (*p*>0.05) for any aperture diameter. The Q values for the female group were more negative than the male group for the anterior surface. However, the results were opposite for the posterior surface.

**Table 3:** Values for male and female groups.

Aperture Diameter (mm)	Q Value (Male)	Q Value (Female)	t	P
**3.0 (Anterior)**	-0.29±0.19	-0.32±0.19	1.969	0.049
**3.0 (Posterior)**	-0.27±0.23	-0.25±0.22	-1.407	0.160
**5.0 (Anterior)**	-0.28±0.19	-0.31±0.19	2.259	0.024
**5.0 (Posterior)**	-0.28±0.22	-0.26±0.22	-1.007	0.314
**7.0 (Anterior)**	-0.29±0.19	-0.31±0.18	2.018	0.044
**7.0 (Posterior)**	-0.27±0.22	-0.26±0.21	-0.797	0.426

### Corneal Q values for different refractive powers

The distribution of Q value based on refractive status is shown in Table 4. The Q values of the anterior surface at 7.0mm and posterior surfaces at 3.0, 5.0 and 7.0mm aperture diameters were statistically significant (*p*<0.05) for the different refractive groups. For the anterior surface, the Q value of the emmetropic group was greater than that of both the myopic and hyperopic group. However, for the posterior surface, the reverse was true.

**Table 4:** Corneal Q values for different refractive powers.

Aperture Diameter (mm)	Myopia (D) SE<-0.50D	Emmetropia (D) -0.50D<SE<+0.50D	Hyperopia(D)SE>+0.50D	F	*p*
**3.0 (Anterior)**	-0.31±0.19	-0.29±0.18	-0.33±0.20	2.863	0.058
**3.0 (Posterior)**	-0.25±0.22	-0.28±0.22	-0.22±0.22	7.155	0.001
**5.0 (Anterior)**	-0.31±0.19	-0.29±0.18	-0.32±0.20	2.941	0.053
**5.0 (Posterior)**	-0.25±0.22	-0.29±0.22	-0.22±0.22	6.814	0.001
**7.0 (Anterior)**	-0.31±0.19	-0.29±0.18	-0.33±0.20	3.411	0.034
**7.0 (Posterior)**	-0.25±0.22	-0.28±0.22	-0.22±0.22	7.112	0.001

### Multiple regression analysis for the corneal Q value and age, sex and refractive power

At the 5.0mm aperture diameter, the corneal Q values of the anterior surface were related to age (B = -0.003, t = -3.760, p<0.01), refractive power (B = -0.013, t = 2.409, p = 0.016) and sex (B = -0.037, t = -2.736, p<0.01). Similarly, the Q values of the posterior surface were related to age (B = 0.002, t = 2.101, p = 0.036), refractive power (B = 0.016, t = 2.025, p = 0.043) and sex (B = 0.047, t = 2.487, p = 0.013).

## Discussion

This study found that the corneal Q values of the anterior surface at 3.0, 5.0 and 7.0mm aperture diameters were -0.29 ± 0.18, -0.28 ± 0.18 and -0.29 ± 0.18, respectively, while some previous studies reported the corneal Q value to be -0.33 (Carney (Australian) [13], Ying (Chinese) [14]), -0.22 (Cheung (Chinese) [15], Scholz (German) [16]), -0.24 (Dubbelman (Dutch) [17]), -0.08 (Horner (Indian) [18]), -0.20 (Fuller (American Caucasian) [6]), -0.26 (Fuller (Africa-American) [6]), -0.346 (Davis (American) [19]), and -0.30 (Zhang (Chinese) [9]). In these studies, the maximum corneal Q value was -0.08, while the minimum value was -0.346. Our results were close to that of Zhang’s [9] study (-0.30). The difference in Q value of our study from previous studies may be due to differences in testing equipment, sample sizes, subject age and race, which we will discuss in details as follows.

### Difference in the test method and equipment

The Bausch & Lomb Orbscan IIz system was utilized in our study and the anterior and posterior surfaces of the cornea were measured separately. Only Ying [14] used the same instrument as we used, and all other studies used different methods. For example: Scholz [16] utilized Tracey iTrace visual function analyzer with integrated EyeSys vista corneal topography, which uses a placido-based videokeratoscope that calculates the Q value by combining the anterior and posterior surfaces as a whole. Carney [13], Cheung [15] and Davis [19] utilized the TMS-I mapping system (topographic modeling system) to collect data. Dubbelman [17] collected the data based on the Topcon SL-45 system, which utilizes the Scheimpflug camera system to measure the depth of focus of axis movement. The EyeSys corneal topography, the PentacamHR system, and the Wavelight-ALLEGRO Topographer were utilized in other studies [6,9,18].

### Difference in the sample size

A large sample size of 1,683 subjects from the Handan Eye Research [12] was included in our study, which could be more representative of the elderly Chinese population. In previous studies, only Zhang's [9] (1052 subjects) and Davis's [19] (1991 subjects) studies had sample sizes over 1000, while others used small sample sizes generally less than 100 subjects.

### Difference in the subject's age

The average age was 53.64 years old in our study and 69.8% of the subjects had the age greater than 50. In the previous studies, the maximum age was 39 years old in Dubbelman’s [17] study. In the Davis's[19] study for Children, the 1,991 subjects’ age range from 6 to 15 years old and the Q value they obtained were -0.346. In Zhang’s [9] study, the subjects were Chinese with average age of 25.4 years old. Although the subjects were younger than ours, they were all adults and the Q value in his study (-0.30) was also close to results we obtained.

### Difference in the subject's race

Purpose of our study was to determine the Q value of the elderly Chinese population. Previous studies by Carney [13], Dubbelman [17] and Davis [19] were performed on Caucasians. Fuller reported the Q values of American Caucasians and African Americans [6]. Horner studied the Q value of Indians [18]. Chan [20] and Cheung [21] also reported the difference for corneal asphericity between Chinese and Caucasians.

The mean corneal Q values for elderly Chinese population were approximately -0.29 for the anterior surface and -0.26 for the posterior surface at 3.0, 5.0 and 7.0mm aperture diameter. Our study found that the mean Q values of the anterior and posterior surface did not change with diameter, which suggested that the cornea was a true quadratic asphere and the asphericity of the cornea could be described by a single Q value. The corneal Q value of the anterior surface trended toward more negative with increasing age. Conversely, the Q value of the posterior surface trended toward less negative with increasing age. The Q values of the anterior surface at 7.0mm and posterior surface at 3.0, 5.0 and 7.0mm aperture diameters were statistically significant (*p*<0.05) for different age groups. This result was different from Zhang’s [7] study which found no significant difference in the Q value between age groups. Our study involves a large sample size and wide age range with an older population, which may provide a dependable reference for future studies.

The corneal Q values of the anterior and posterior surface in our study were not normally distributed. Instead, it skewed to the left (negative). This result agreed with previous findings from Holmes-Higgin (-0.01~-0.81) [22], Townsley (-0.01~-0.64) [23], Eghbali (-0.11~-0.26) [24], Mandell (-0.04~-0.72) [25], and Budak (-0.11~-0.33) [26]. Also, González-Méijome [27] reported that the Q values of the anterior surface were between 0 and -1, indicating a prolate ellipse. However, some other studies [16] reported that the Q values were between 1 and -1, but the distribution of the Q value were not discussed nor presented in these studies.

Our study found a correlation between the corneal Q value and sex. Similar conclusions were obtained by Carney [13], Scholz [16], Dubbelman [17], and Chan [20]. In our study, the female group had a higher negative Q value than that of the male group for the anterior surface, indicating that females tended to have an anterior surface with higher asphericity. In contrast, the opposite was true for the posterior surface. Though Fuller [6] and Cheung [15] found the corneal Q values were irrelevant to the sex, the sample sizes in their studies were much smaller and may not have had the power to detect a small difference.

In addition, our study found that mean Q values were different depending on refractive power. For the anterior surface, the Q value was greater in the emmetropic group compared to the myopic and hyperopic group. Conversely, for the posterior surface, the trend was opposite. The Q values of the posterior surfaces at 3.0, 5.0 and 7.0 mm aperture diameters were statistically significant for the different refractive powers (*p*<0.05).

At the same time, there are some limitations shown in our study. First, in this study, we did not include the subjects that were younger than 30 years old. Therefore, there were no analysis conducted on the differences of the Q value among children, youth and young adults, or whether the Q value changes at the age of puberty while it is quite stable for adults. Second, during the process of measuring corneal topography, older people and people with poor visions due to cataract were usually unable to cooperate. This could cause a selection bias in sample collection, which led to a decrease in the number of high myopia. As a result, the prevalence of high myopia (<-5.00D) in our study (1.1%) was lower than that reported in the Handan Eye Study (1.8%) [28].

As a summary, our study found the corneal Q values of the elderly Chinese were different from previous studies involving European and American populations. Age, sex and refractive power seemed to be correlated with the Q value as well. The results may provide a useful reference for designing visual optical products and future study of the optical properties of the human eye.
